# Socioeconomic disparities in health-related quality of life and healthcare use in the last year of life of patients with advanced cancer: longitudinal results from the eQuiPe study

**DOI:** 10.1007/s00520-025-09309-9

**Published:** 2025-03-11

**Authors:** M. A. J. Versluis, Y. M. van der Linden, S. Oerlemans, D. W. Sommeijer, W. K. de Jong, A. Baars, T. J. Smilde, A. van der Padt-Pruijsten, L. V. van de Poll-Franse, N. J. H. Raijmakers

**Affiliations:** 1 Graduate School of Social and Behavioral Sciences, Tilburg University, PO Box 90153, 5000 LE Tilburg, The Netherlands; 2 Research and Development, Netherlands Comprehensive Cancer Organisation (IKNL), Utrecht, The Netherlands; 3 Centre of Expertise in Palliative Care, Leiden University Medical Centre, Leiden, The Netherlands; 4 Department of Radiotherapy, Leiden University Medical Centre, Leiden, The Netherlands; 5 Department of Internal Medicine, Flevoziekenhuis, Almere, The Netherlands; 6 Department of Pulmonology, University Medical Center Utrecht, Utrecht, The Netherlands; 7 Department of Internal Medicine, Hospital Gelderse Vallei, Ede, The Netherlands; 8 Department of Medical Oncology, Jeroen Bosch Hospital, SHertogenbosch, The Netherlands; 9 Department of Internal Medicine, Maasstad Hospital, Rotterdam, The Netherlands; 10 Department of Medical and Clinical Psychology, Corps – Center for Research on Psychology in Somatic Diseases,AQ, Tilburg University, Tilburg, The Netherlands; 11 Department of Psychosocial Research and Epidemiology, The Netherlands Cancer Institute (NKI), Amsterdam, The Netherlands

**Keywords:** Socioeconomic position, Health equity, Quality of life, Healthcare use, Advanced cancer, Palliative care

## Abstract

**Purpose:**

To examine socioeconomic disparities in health-related quality of life (HRQoL) and healthcare use during the last year of life of patients with advanced cancer.

**Methods:**

Data was used from a prospective, longitudinal, multicenter, observational study of patients with advanced cancer in forty Dutch hospitals (eQuiPe). Adult patients with stage IV cancer completed 3-monthly questionnaires until death. Socioeconomic position (SEP) was defined as estimated income on street-level. Mixed-effects regression analysis was used to identify associated factors.

**Results:**

A total of 639 patients were included, 14% with a lower SEP, 59% medium SEP and 28% higher SEP. Patients with a lower SEP were more often lower educated (40% vs. 18%, *p* < 0.001) and less often reported to have a partner (61% vs. 90%, *p* < 0.001) than those with a higher SEP. In the last year of life, patients with lower SEP were more likely to experience disease-related financial difficulties than those with higher SEP (28% vs. 12%, p = 0.001; β 8.2, 95%CI 2.9–13.3). No significant associations were found between SEP and HRQoL, hospital admissions or emergency department admissions. Although, patients with lower SEP had more frequent (≥ 5 per month) interactions with healthcare professionals than patients with higher SEP in the last year of life (OR 1.9, 95%CI 1.0–3.5).

**Conclusion:**

Some socioeconomic disparities are present during the last year of life of patients with advanced cancer. It is important for clinicians to be aware of the greater financial impact and higher healthcare utilization in patients with a lower SEP to ensure equitable end-of-life care.

**Supplementary information:**

The online version contains supplementary material available at 10.1007/s00520-025-09309-9.

## Introduction

One in five cancer diagnoses is metastatic at the time of primary diagnosis, and an even greater proportion of patients will develop metastases at a later stage [1]. Although cancer treatments and screening programs are improving, median survival for most patients with advanced cancer remains limited [2]. For patients with advanced cancer, curative treatments are often not available, and care and treatment are focused on quality of life (QoL). QoL can be improved by palliative care, through early identification, assessment and treatment of pain and other physical, psychosocial, and spiritual problems [3]. Equal access to palliative care is paramount. However, differences in patients’ social surroundings and economic situations lead to inequalities in cancer care. These socioeconomic inequalities reflect disparities between patients of different social and economic position, which is often a complex and multifaceted concept typically measured through income, education or occupation [4]. Known socioeconomic inequalities in cancer care are delayed diagnosis in patients with low socioeconomic position (SEP), differences in treatment choices between patients depending on their levels of SEP, more multimorbidity in patients with low SEP and higher cancer mortality rates in patients with low SEP [5–10]. Also, within palliative care, patients with low SEP appear to face a disproportionate burden as they experience higher symptom burden, higher levels of anxiety and depression, and generally have a lower QoL than patients with higher SEP [11, 12]. This suggests that patients with lower SEP might need more care and support than those with higher SEP. It is therefore remarkable that patients with lower SEP are less likely to see a medical specialist, have less access to hospice care, and are less likely to see a hospital-based palliative care team [13–15]. This suggests that despite the greater care needs of patients with lower SEP, their healthcare use is lower.

Because most studies on socioeconomic inequalities in palliative care were cross-sectional, cancer-specific or focused on a particular type of care, little is known about SEP differences in healthcare use and QoL in the last year of life. QoL is known to change over the course of the disease [16, 17] and therefore, SEP differences may also change. A deeper understanding of these socioeconomic differences in QoL and healthcare use among patients with advanced cancer in the last year of life is crucial to address appropriate and equitable access to care for this group of patients. Therefore, the aim of this study was to examine variations in healthcare use and QoL in the last year of life of patients with advanced cancer according to their socioeconomic position. Understanding these inequalities can inform health policy and resource allocation to ensure that vulnerable populations receive the care and support they need to navigate the complexities of advanced cancer care.

## Materials and methods

### Study design

Longitudinal data were used from the Dutch prospective, longitudinal, multicenter, observational study on the experienced quality of life and quality of care in patients with advanced cancer and their relatives (eQuiPe study), of which the details have been reported elsewhere [18]. In short, patients were invited by their treating physician from one of the forty participating hospitals or self-registered between November 2017 and March 2020. After signing an informed consent, patients received a baseline questionnaire followed by a three-monthly follow-up questionnaire until death or loss to follow-up. All questionnaires were completed via the Patient Reported Outcomes Following Initial Treatment and Long-term Evaluation of Survivorship (PROFILES) registry [19] and linked to the Netherlands Cancer Registry (NCR). The eQuiPe study was exempted from medical ethical review by the Medical Research Ethics Committee of the Antoni van Leeuwenhoek Hospital (METC17.1491) in accordance with the Dutch Medical Research Involving Human Subjects Act. The study is registered on the 30th of November 2017 in the Netherlands Trial Register as NL6408 (https://onderzoekmetmensen.nl/).

### Study population

All patients diagnosed with stage IV cancer, aged over 18 years, were eligible for inclusion. As the overall aim of the eQuiPe study was to assess the QoL and quality of care in the last year of life of patients with advanced cancer, additional inclusion criteria were added for patients with breast and prostate cancer to prevent the inclusion of patients with a relatively good prognosis. Patients with breast cancer were eligible if metastases were found in multiple organ systems, and patients with prostate cancer were eligible if their cancer was castrate resistant. For this analysis, only patients who had died during the study period were selected (data cut-off January 2023).

### Measurements

#### Socioeconomic position

SEP was defined as estimated income at street level and was obtained from the NCR [20]. Median household income was estimated using the patient’s six-digit postal code in 2016, covering approximately seventeen households. The NCR divided SEP into nine percentiles based on the general income levels of the Dutch population. SEP was then categorized into low (first to third percentile), medium (fourth to sixth percentile) and high (seventh to ninth percentile).

#### Health-related quality of life

Health-related quality of life (HRQoL) was measured using the European Organization for Research and Treatment of Cancer Quality of Life questionnaire (EORTC QLQ-C30) [19, 21], which is available online [22]. The EORTC QLQ-C30 consists of a global health status, five functioning subscales (physical, emotional, role, cognitive and social), three symptom subscales (fatigue, nausea/vomiting, and pain), five individual symptom items (insomnia, dyspnea, constipation, diarrhea and loss of appetite) and one item on disease-related financial difficulties. All scores were linearly transformed to a 0–100 score, with higher scores indicating better functioning and higher symptom burden. In addition, the percentage of patients with clinically relevant problems were calculated using the cut-off values described by Giesinger et al. [23] for all HRQoL functioning (except for the global health status as no threshold is available) and symptom scales to provide more interpretable results for clinical practice.

#### Healthcare use

Healthcare use was measured using a self-composed questionnaire. Patients were asked how often and which healthcare professional they had contacted in the previous month. Healthcare professionals included in the response options were: general practitioner, medical specialist, nurse specialist, home care nurse, palliative care team consultant, psychologist, sexologist, social worker, spiritual caregiver, physiotherapist, ergo therapist, dietician and other. Consequently, the number of contacts were summed up to a total number of healthcare contacts in the previous month. To use the same model for all healthcare use variables and to simplify the interpretation, the number of healthcare contacts was then dichotomized using the median number of total healthcare contacts of the total population as a cut-off (more or less than 5 contacts). Hospital admission (“Have you been hospitalized in the past month?”) and emergency department admission (“Have you visited the emergency department in the past month?”) were both assessed with a single-item question.

#### Sociodemographic and clinical characteristics

Date of birth, marital status, religion, and level of education were self-reported in the baseline questionnaire. Age was defined as age at death and calculated as years from date of birth to date of death. Religion was a single item asking patients to what religion they belonged, which was categorized as religious or not. Education was categorized into three categories (low-medium–high) according to the guidelines of the International Standard Classification of Education. Comorbidity was measured at baseline using the Self-administered Comorbidity Questionnaire (SCQ) [24]. Primary cancer type and date of death were obtained from the NCR. Time to death was defined as time (months) between completion of the questionnaire and date of death.

### Statistical analysis

Descriptive analyses were used to describe the sociodemographic and clinical characteristics of the general population, and ANOVA and chi-squared tests were used to univariably examine differences in sociodemographic and clinical characteristics between SEP levels. Descriptive analyses were used to observe the changes in HRQoL and symptoms over time, stratified for SEP, and chi-squared tests were used to assess the cross-sectional differences in percentage of patients with clinically relevant problems between SEP levels using the questionnaire that was completed closest to a patient’s death. Linear mixed-effects modelling was used to examine the longitudinal association between SEP and all HRQoL functioning and symptom scales and logistic mixed-effects modelling was used to examine the longitudinal association between SEP and healthcare use. Statistical significance was set at *p* < 0.05. All statistical analyses were performed using STATA 17.0.

## Results

**Fig. 1:**
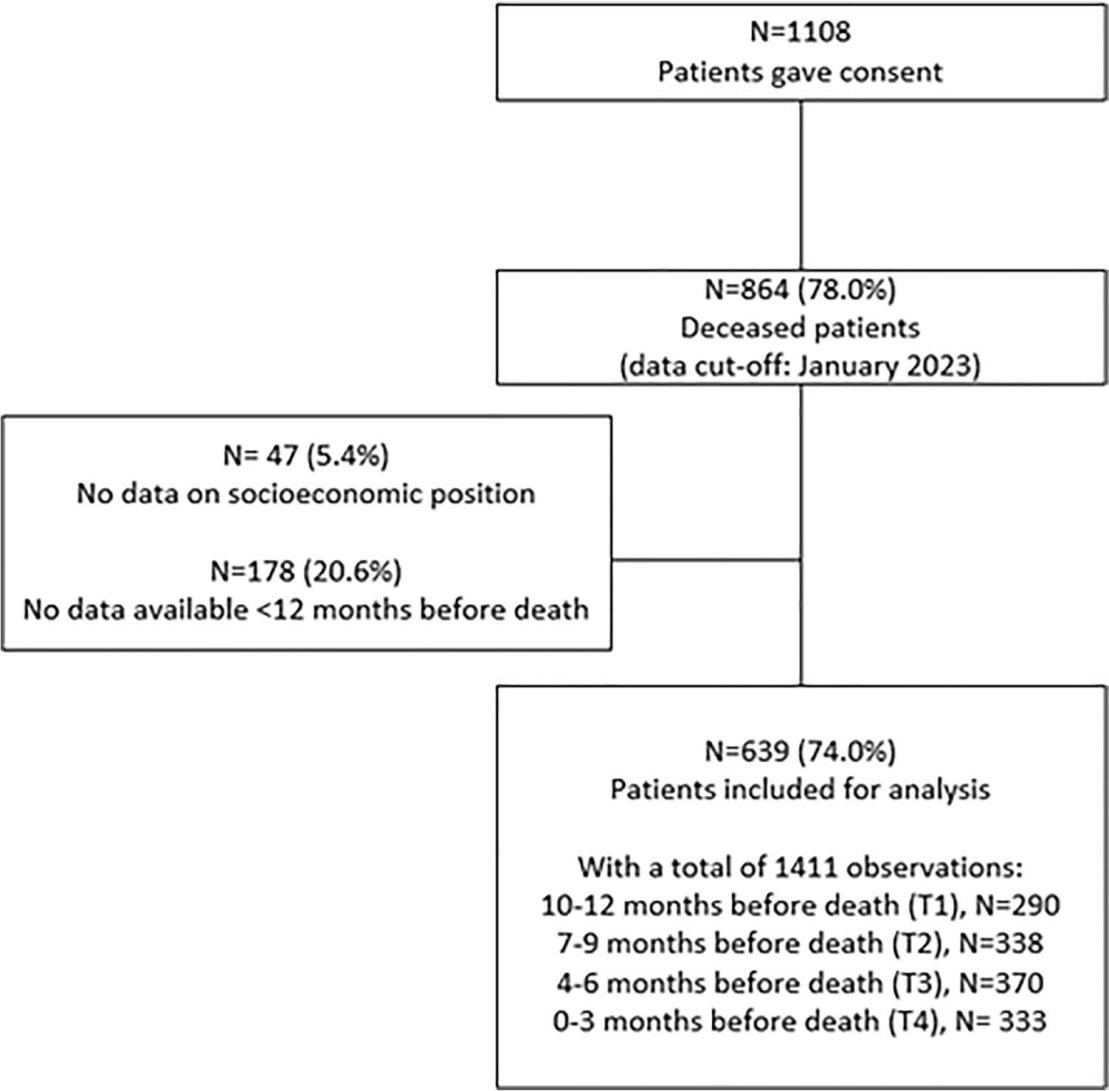
Flowchart of the inclusion of patients

A total of 639 patients were included in the analysis (Fig. 1). Mean number of questionnaires completed in the last year of life was 2 (range 1–5). Mean age at time of death was 67 (SD 10) and 47% was female (Table 1). Approximately one-fifth (18%) had metastasis at primary diagnosis and the three most reported cancer types were lung (28%), colorectal (21%) and breast (11%).

**Table 1:** Socio-demographic and clinical characteristics of 639 patients with advanced cancer

	Total population (N = 639) N (%)	Low SEP (N = 88, 14%) N (%)	Medium SEP (N = 374, 59%) N (%)	High SEP (N = 177, 28%) N (%)	*p*-value
Age (mean (SD))	67 (10)	68 (9)	67 (10)	66 (10)	0.155
Gender					
Male	333 (52)	42 (48)	202 (54)	90 (51)	0.257
Female	299 (47)	46 (52)	167 (45)	87 (49)	
Education^a^					** < 0.001**
Low	200 (31)	35 (40)	135 (36)	31 (18)	
Medium	257 (40)	34 (39)	146 (39)	77 (44)	
High	172 (27)	18 (20)	86 (23)	69 (39)	
Partner (yes)	517 (81)	54 (61)	306 (82)	159 (90)	** < 0.001**
Religious(yes)	428 (67)	65 (74)	246 (66)	117 (66)	0.196
Comorbidities (yes)					0.366
Yes	409 (64)	63 (72)	237 (63)	109 (62)	
No	189 (30)	22 (25)	109 (29)	58 (33)	
Missing	41 (6)	3 (3)	28 (7)	10 (6)	
Primary cancer type					0.166
Lung	182 (28)	34 (39)	110 (29)	38 (21)	
Colorectal	134 (21)	14 (16)	80 (21)	40 (23)	
Breast	73 (11)	9 (10)	38 (10)	26 (15)	
Prostate	57 (9)	6 (7)	31 (8)	20 (11)	
Stomach/Esophagus	31 (5)	4 (5)	17 (5)	12 (7)	
Gynecologic	15 (2)	1 (1)	12 (3)	5 (3)	
Pancreas	15 (2)	1 (1)	10 (3)	7 (4)	
Urinary tract	14 (2)	-	11 (3)	7 (4)	
Other	116 (18)	19 (22)	163 (7)	22 (12)	
Metastasis at primary diagnosis (yes)	117 (18)	12 (14)	68 (19)	37 (22)	0.322
Years since diagnosis (mean (SD))	4 (4)	4 (5)	3 (3)	4 (4)	0.143

^*a*^* Education was defined as low* (no education, pre-primary education, primary education, lower secondary education, compulsory education, initial vocational education), medium (upper secondary general education, basic vocational education, secondary vocational education, post-secondary education) and high (specialized vocational education, university/college education, (post)doctorate and equivalent degrees)^*b*^* Missings did not exceed* > *5%, unless presented otherwise*

Fourteen percent of patients had a low SEP, 59% medium SEP and 28% high SEP. Patients with low SEP had fewer years of education (40% vs. 18%, *p* < 0.001) and less often reported to have a partner (61% vs. 90%, *p* < 0.001) compared to patients with high SEP.

### HRQoL

Descriptive analyses showed no visible differences in trajectories of HRQoL or symptoms between levels of SEP (Supplementary information 1). Univariable analyses showed no significant differences in experiencing clinically relevant problems in functioning and symptoms between levels of SEP, except for perceived financial difficulties. The percentage of patients experiencing financial difficulties was significantly higher in patients with low SEP compared to patients with high SEP (28% vs. 12%, *p* = 0.001) (Table 2). Multivariable mixed-effects linear regression analyses confirmed that patients with low SEP were more likely to have greater perceived financial difficulties than patients with high SEP (β 8.2, 95% CI 2.9–13.3) (Supplementary information 2).

**Table 2:** Percentage of patients with clinically relevant problems^a^ using the observation closest to death for each patient stratified by SEP (*n* = 639)

	Low SEP (*n* = 88) N (%)	Medium SEP (*n* = 374) N (%)	High SEP (*n* = 177) N (%)	*p*-value
Mean time to death^b^	5 (SD 4)	4 (SD 3)	5 (SD 3)	0.603
Functioning scale				
Physical	65 (74)	271 (72)	131 (74)	0.771
Emotional	42 (48)	141 (38)	63 (36)	0.094
Social	23 (26)	87 (23)	47 (27)	0.631
Cognitive	27 (31)	144 (39)	58 (33)	0.254
Role	45 (51)	163 (44)	90 (51)	0.151
Symptom burden				
Fatigue	55 (63)	198 (53)	97 (55)	0.159
Pain	48 (55)	186 (50)	89 (50)	0.594
Dyspnea	46 (52)	222 (59)	108 (61)	0.398
Insomnia	29 (33)	101 (27)	54 (31)	0.454
Loss of appetite	19 (22)	97 (26)	46 (26)	0.756
Nausea	39 (44)	156 (42)	81 (46)	0.615
Constipation	13 (15)	41 (11)	16 (9)	0.300
Diarrhea	30 (34)	103 (28)	55 (31)	0.366
Financial difficulties	**25 (28)**	**90 (24)**	**21 (12)**	**0.001**

^*a*^* Clinically relevant problems are present when functioning scores are below the cut-off values and symptom scores are above the cut-off values defined by Giesinger *et al. [24]^*b*^* Mean time to death is presented in months*

### Healthcare use

The median number of total healthcare contacts in the last year of life ranged from 6 to 8 in low SEP, 5 to 7 in medium SEP and 4 to 6 in high SEP (Supplementary information 3). Multivariable mixed-effects logistic regression model showed that patients with low SEP were more likely to have ≥ 5 healthcare contacts than patients with high SEP (OR 1.88, 95% CI 1.03–3.45) (Table 3). Having ≥ 5 healthcare contacts was also associated with time to death (OR 0.96, 0.91–1.00), female sex (OR 1.87, 1.22–2.87), colorectal vs. lung cancer (OR 2.17, 1.27–3.73) and having comorbidities (OR 1.50, 1.01–2.22). The most commonly reported healthcare professional that patients had contacted within their last year of life was the medical specialist, for both low SEP (78%) and high SEP patients (74%) (Supplementary information 4).

**Table 3:** Mixed-effects logistic regression model to assess the association between healthcare use and SEP in the last year of life of patients with advanced cancer

	≥ 5 visits (*n* = 573)		Hospital admission (*n* = 546)		ED admission^a^ (*n* = 546)	
	OR	95% CI	OR	95% CI	OR	95% CI
Time until death (months)	**0.96**	**0.91–1.00**	**0.91**	**0.87–0.97**	**0.89**	**0.84–0.95**
SEP						
High	Ref		Ref		Ref	
Medium	1.37	0.90–2.07	1.12	0.69–1.83	0.96	0.60–1.56
Low	**1.88**	**1.03–3.45**	1.70	0.87–3.31	1.88	0.96–3.66
Age at death (years)	0.99	0.97–1.01	**0.96**	**0.94–0.98**	**0.96**	**0.94–0.98**
Gender						
Male	Ref		Ref		Ref	
Female	**1.87**	**1.22–2.87**	0.86	0.54–1.37	0.78	0.48–1.26
Education						
High	Ref		Ref		Ref	
Medium	1.36	0.87–2.12	0.82	0.50–1.36	0.89	0.54–1.47
Low	1.32	0.81–2.15	1.04	0.60–1.80	1.00	0.57–1.73
Partner (yes)	0.69	0.43–1.11	1.39	0.80–2.42	**2.23**	**1.20–4.16**
Religious (yes)	1.35	0.92–1.98	1.30	0.83–2.02	1.35	0.86–2.12
Cancer type						
Lung	Ref		Ref		Ref	
Colorectal	**2.17**	**1.27–3.73**	0.99	0.54–1.84	0.84	0.45–1.57
Breast	1.11	0.58–2.09	0.79	0.38–1.65	0.74	0.35–1.56
Prostate	1.31	0.66–2.59	0.61	0.26–1.43	0.90	0.41–2.00
Stomach/Esophagus	1.08	0.42–2.78	1.63	0.57–4.68	0.76	0.23–2.51
Pancreas	3.38	0.97–11.76	1.45	0.43–4.92	1.43	0.42–4.92
Gynecologic	1.48	0.52–4.25	0.39	0.09–1.74	0.32	0.06–1.69
Urinary tract	0.75	0.24–2.35	0.36	0.06–2.02	0.83	0.19–3.57
Other	1.17	0.69–1.99	1.45	0.81–2.59	1.09	0.60–1.97
Metastasis at primary diagnosis (yes)	0.98	0.65–1.47	1.32	0.82–2.12	1.03	0.63–1.71
Comorbidities (yes)	**1.50**	**1.01–2.22**	1.44	0.92–2.27	1.47	0.93–2.33

^*a*^* ED, *emergency department

### Hospital and emergency department admission

**Fig. 2:**
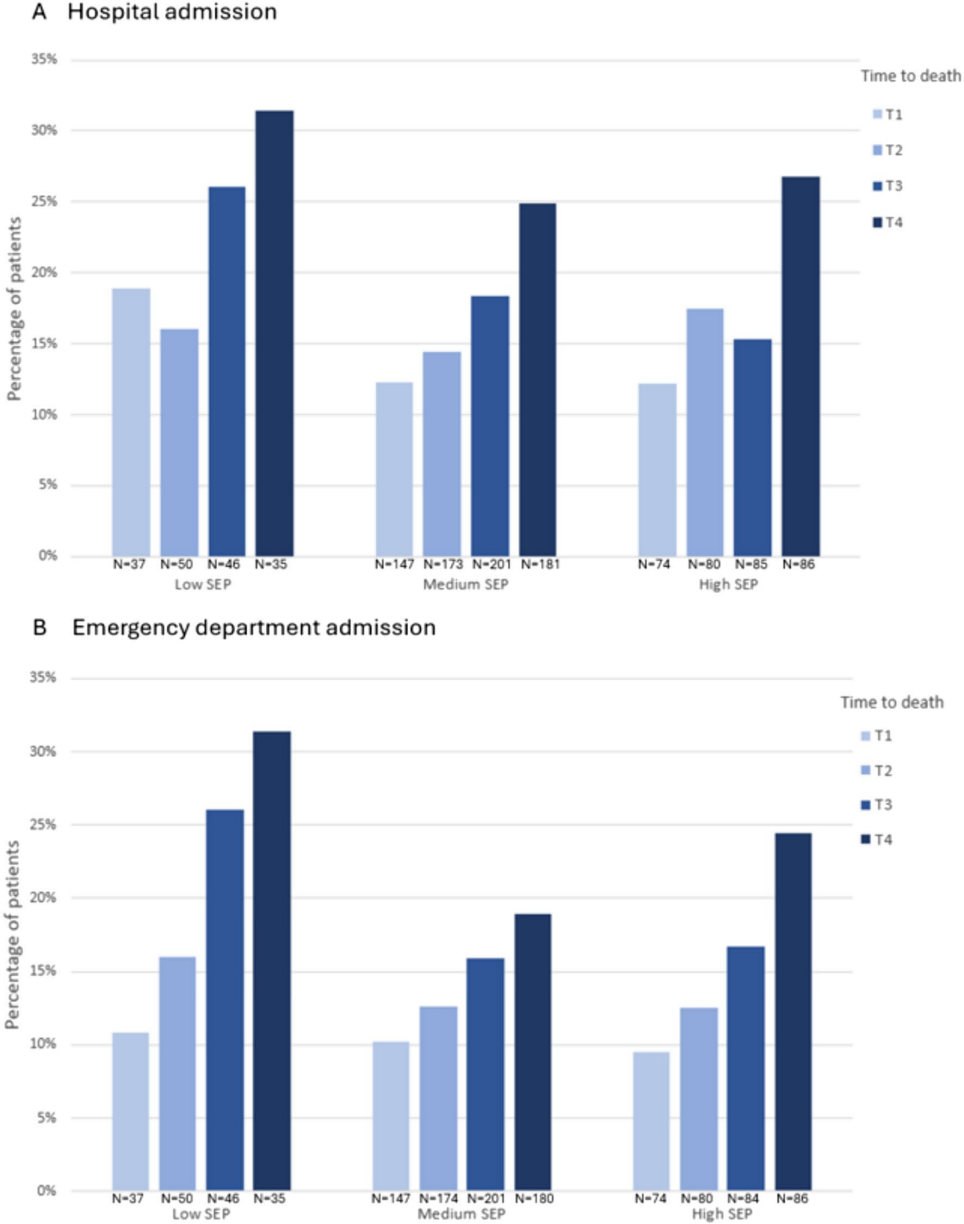
Number of patients who visited the hospital or emergency department (ED) during the past month stratified by SEP and visualized per time category

The proportion of patients with low SEP who were admitted to the hospital was 19% 10–12 months before death and 31% in the last 3 months of life, for patients with high SEP, this was respectively 12% and 27% (Fig. 2a). The proportion of patients who were admitted to the emergency department was 11% 10–12 months before death and 31% in the last 3 months of life for patients with low SEP and respectively 9% and 24% for patients with high SEP (Fig. 2b). Multivariable mixed-effects logistic regression analyses showed that neither admission to the hospital nor emergency department was associated with SEP (Table 3). The likelihood of being admitted to the hospital was lower with time further away from death (OR 0.91, 95% CI 0.87–0.97) and in older patients (OR 0.96, 0.94–0.98). The likelihood of being admitted to the emergency department was also lower with time further away from death (OR 0.89, 0.84–0.95) and in older patients (OR 0.96, 0.94–0.98). Patients who had a partner were more likely to be admitted to the emergency department (OR 2.23, 1.20–4.16).

## Discussion

We found no socioeconomic differences in HRQoL and symptom burden in the last year of life of patients with advanced cancer, except for disease-related financial difficulties. Patients with lower SEP more often experienced financial difficulties during their last year of life compared to patients with high SEP. Patients with lower SEP were more likely to have multiple contacts with healthcare professionals per month, compared to high SEP patients. No socioeconomic differences were found in the likelihood to be admitted to the hospital or the emergency department. Independent of SEP, healthcare use increased towards the end of life.

Our finding that no socioeconomic differences in HRQoL and symptom burden were present was surprisingly. Previous studies in patients with advanced cancer showed that low SEP was associated with poorer quality of life outcomes [12, 25]. Also, a recent analysis in a similar setting but including cancer survivors showed that survivors with low SEP more frequently reported impaired HRQoL than survivors with medium SEP [26]. This study also described similar trends in matched cancer-free population. However, to our knowledge, no study has been conducted before assessing this association specifically in the last year of life of patients with advanced cancer or using longitudinal data. Therefore, our results are hard to compare with other study results and must be confirmed with future research. Even though our results indicate that the trajectory of HRQoL in the last year of life may be similar for patients across all levels of SEP, it is well known that patients with low SEP are likely to experience more psychological distress [27]. Clinicians should remain aware of these possible inequalities to assure equitable, high quality of care for all patients.

Our results do show that patients with low SEP have greater perceived financial difficulties in the last year of life compared to patients with high SEP. This finding is consistent with another Dutch study showing that patients with low SEP have a 13% higher healthcare expenditure than high SEP patients [28]. However, this study also shows that the association between SEP and healthcare expenditure becomes non-significant after adjusting for the overall health status in the model. This finding suggests that healthcare costs are primarily driven by health status rather than SEP directly, though patients with low SEP often experience poorer health and consequently require more intensive healthcare services. In addition, another Dutch study showed that living in a low SEP neighborhood is also associated with higher healthcare costs, which they also link to worse overall health in that area [29]. A UK study showed that despite a system of free healthcare, patients with low SEP have higher end-of-life treatment costs than patients with high SEP [30]. However, not only healthcare specific factors may play a role in the overall financial burden of these patients. There may be other organizational or logistical barriers, such as travel costs and loss of income. Although more research is needed to explore the factors associated with greater financial difficulties, clinicians should already be aware of its existence and support their patients in arranging (financial) support if needed. Clinicians should facilitate referrals to social workers who can help patients with optimal support.

Patients with low SEP were 88% more likely to have more than five contacts with any healthcare professional in their last year of life than those with high SEP. The reasons behind these visits were not measured in this study. Patients with low SEP may have worse overall health and may have more difficulties adjusting to their diagnosis [31]. Also, patients with low SEP are more prone to psychological distress and more often lack a social support network compared to patients with high SEP [27, 32], which is also associated with a worse mental health status and worse physical and psychological adjustment to cancer [33]. Moreover, patients with low SEP often have lower health literacy. Patients with lower health literacy skills often experience more difficulties navigating through the healthcare system and a limited understanding of their cancer symptoms resulting in a higher need for (emotional) support from their healthcare providers [34, 35]. This, together with the general higher care need and limited social support, may lead to a higher tendency to seek professional help as seen in our study results. Designating a case manager as primary point of contact could help streamline access to services and provide consistent support for these patients. However, higher healthcare use may not always be the result of a higher care need and may also indicate overtreatment. Some studies showed an increased likelihood of receiving aggressive end-of-life care and a decreased likelihood of choosing non-treatment options in patients with low SEP compared to patients with high SEP [36, 37].

While our study found no association between SEP and rates of hospital admission or emergency department visits in the last year of life, these findings diverge from existing literature. Further research with a larger sample of patients with low SEP is needed to validate, given the inconsistent findings across previous studies. A recent systematic review reported that, independent of diagnosis, low SEP is associated with more hospital visits in the last three months of life [38]. In addition, a Taiwanese study also showed that patients with low SEP had more emergency department visits and intensive care unit admissions than low SEP cancer patients [39]. In contrary, a recent Norwegian study showed that a low income was associated with fewer hospitalizations in the last year of life of patients with advanced cancer [40]. Most likely, the presence of socioeconomic disparities in healthcare use is partially depending on the organization of healthcare and health insurance in a country. For example, in The Netherlands, everyone is obliged to have a basic health insurance, which covers most of the oncological costs. Moreover, as a densely populated country, Dutch medical oncology patients have an average travel time of only 30 min to reach one of the 65 hospitals [41]. Therefore, geographical location may not impact the Dutch oncology patients as much as patients living in less densely populated countries.

At last, our study showed that the healthcare use increased towards death, independently of the level of SEP. This increase in healthcare use in the last year of life has been reported in previous studies. A Scottish study of 274,048 decedents reported an increase in secondary care use, with a sharp increase in the last three months of life [42]. Also in the UK, healthcare use and costs increased in the last year of life [43]. This increase in time may be consistent with the previously reported decrease in quality of life and increase in symptom burden in the last year of life of advanced cancer patients, suggesting an increasing need for care towards death [44]. However, a distinction should be made between appropriate and potentially inappropriate end-of-life care. For example, high numbers of hospital admissions and emergency department visits in the last month of life are indicators of potentially inappropriate end-of-life care [45]. This study shows that healthcare use increases in the last year of life of patients with advanced cancer and highlights the need for timely access to (hospital-based) palliative care and the need for advanced care planning as this can prevent potentially inappropriate end-of-life care [46].

### Strengths and limitations

This unique prospective longitudinal observational multicenter study allowed us to assess socioeconomic differences in the last year of life of patients with advanced cancer. The eQuiPe study had an overall response rate of 65% and despite loss of follow-up 43% of patients were able to complete a questionnaire in their last three months of life. However, there are still some limitations that need to be addressed. Firstly, it is well known that patients with a better health status are more likely to participate in survey studies resulting in selection bias [47]. Besides gatekeeping, the overall worse health status in patients with low SEP may also be why this group is relatively underrepresented in this study (14%). The underrepresentation of patients with low SEP and the possible selection of more healthy patients may have led to less prominent differences in HRQoL and healthcare use between SEP groups. Future research with more representative sampling is needed to further examine these relationships. The disparities found in this study may, therefore, also be an underestimate of reality. Second, the follow-up period of this study partially took place during the COVID-19 pandemic. However, previous studies that have shown the oncological care in advanced cancer patients were hardly affected by the pandemic in The Netherlands [48, 49]. Third, healthcare use was self-reported and may have been over- or underestimated. Finally, SEP was estimated as household income on street-level and may not be accurate for an individual patient. Moreover, SEP is a complex and multifaceted construct that extends beyond individual income. Future research should consider several factors (combined) influencing SEP, like education, occupation, ethnicity, and income.

## Conclusion

In the last year of life of patients with advanced cancer, no socioeconomic disparities were found in HRQoL except for perceived financial difficulties. Patients with low SEP had greater perceived financial difficulties. Clinicians should be aware of this and facilitate referrals to social workers who can help them navigate through the different options for (financial) support to ensure equitable end-of-life care. Patients with low SEP also had more contacts with healthcare professionals. Designating a case manager as primary point of contact could help streamline access to services and provide consistent support for these patients. Independent of SEP, hospital and emergency department admissions increased in the last year of life highlighting the importance of advanced care planning to prevent potentially inappropriate end-of-life care.

## Supplementary information

Below is the link to the electronic supplementary material.

ESM 1 (DOCX 200 KB)

## Data Availability

Data from the eQuiPe study is available through the Questacy (DDI 3.x XML) and can be accessed at the PROFILES registry (www.profilesregistry.nl). Quality guidelines developed by Data Archiving and Networking Services (DANS) and reported in the “Data Seal of Approval” document were followed for optimal long-term warehousing and dissemination (www.datasealofapproval.org).
