# Supplementary material for: Socioeconomic disparities in health-related quality of life and healthcare use in the last year of life of patients with advanced cancer: longitudinal results from the eQuiPe study
Source: Support Care Cancer. 2025 Mar 11;33(4):265. doi: 10.1007/s00520-025-09309-9 (PMC11897117; doi:10.1007/s00520-025-09309-9)
Supplement: Supplementary file 1 — (DOCX 200 KB) [file 520_2025_9309_MOESM1_ESM.docx]

**Supplementary materials**

Supplement 1: Trajectories of health-related quality of life and symptom burden in patients with advanced cancer stratified for socioeconomic position.


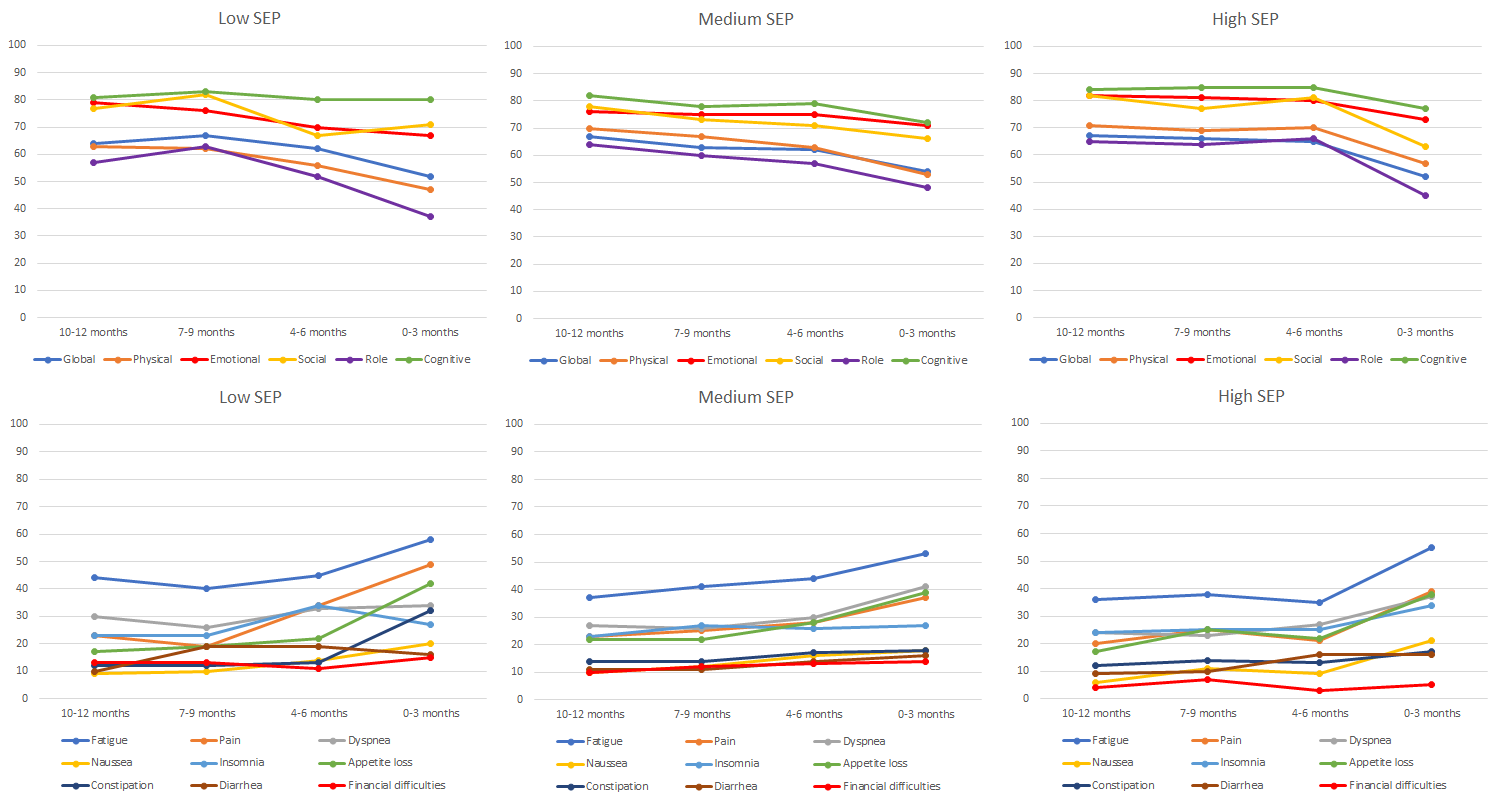


**Supplement 2:** Mixed-effects analysis to assess the association between quality of life and SEP in the last year of life of patients with advanced cancer.

| **Tabel 1: Mixed-effects linear regression model for Quality of life: functioning scores (0-100)** | | | | | | | | | |
| --- | --- | --- | --- | --- | --- | --- | --- | --- | --- |
|  | Physical  (n=552) | | Emotional  (n=551) | | Social  (n=551) | | Cognitive  (n=551) | Role  (n=551) | Global  (n=553) |
|  | β | 95% CI | β | 95% CI | β | 95% CI | β 95% CI | β 95% CI | β 95% CI |
|  |  |  |  |  |  |  |  |  |  |
| **Time until death** *(months)* | **2.0** | **0.7 ; 2.3** | **0.8** | **0.5 ; 1.1** | **1.5** | **1.1 ; 1.9** | **1.1 0.8 ; 1.4** | **2.1 1.7 ; 2.6** | **1.5 1.2 ; 1.8** |
| **SEP** |  |  |  |  |  |  |  |  |  |
| High | Ref |  | Ref |  | Ref |  | Ref | Ref | Ref |
| *Medium* | -1.8 | -6.0 ; 2.4 | -0.8 | -4.4 ; 2.9 | -3.3 | -7.5 ; 0.8 | -3.6 -7.3 ; 0.2 | 0.1 -5.0 ; 5.3 | -0.4 -3.8 ; 2.9 |
| *Low* | -3.8 | -9.8 ; 2.2 | -1.4 | -6.7 ; 3.9 | 0.0 | -6.0 ; 6.0 | 1.2 -4.3 ; 6.6 | -1.6 -9.0 ; 5.8 | 1.3 -3.6 ; 6.2 |

| **Tabel 2: Mixed-effects linear regression model for Quality of life: symptom burden scores (0-100)** | | | | | | | | | | | | |
| --- | --- | --- | --- | --- | --- | --- | --- | --- | --- | --- | --- | --- |
|  | Fatigue  (n=551) | | Pain  (n=550) | | Insomnia  (n=551) | | Dyspnea  (n=550) | Appetite loss  (n=549) | Nausea  (n=550) | Constipation  (n=550) | Diarrhea  (n=548) | Financial  (n=552) |
|  | β | 95% CI | β | 95% CI | β | 95% CI | β 95% CI | β 95% CI | β 95% CI | β 95% CI | β 95% CI | β 95% CI |
|  |  |  |  |  |  |  |  |  |  |  |  |  |
| **Time until death** *(months)* | **-1.9** | **-2.2 ; -1.5** | **-2.0** | **-2.4 ; -1.6** | **-0.5** | **-0.9 ; -0.0** | **-1.4 -1.9 ; -1.0** | **-2.1 -2.6 ; -1.6** | **-1.1 -1.5 ; -0.8** | **-0.9 -1.3 ; -0.5** | **-0.8 -1.2 ; -0.4** | -0.1 -0.4 ; 0.2 |
| **SEP** |  |  |  |  |  |  |  |  |  |  |  |  |
| High | Ref |  | Ref |  | Ref |  | Ref | Ref | Ref | Ref | Ref | Ref |
| *Medium* | 1.2 | -3.1 ; 5.5 | 1.9 | -2.7 ; 6.5 | -2.8 | -7.7 ; 2.1 | -0.8 -5.9 ; 4.2 | 1.6 -3.3 ; 6.6 | 1.9 -1.4 ; 5.2 | 0.6 -3.3 ; 4.4 | -0.7 -4.6 ; 3.1 | **5.1 1.5 ; 8.7** |
| *Low* | 2.2 | -4.0 ; 8.3 | 3.1 | -3.5 ; 9.6 | -1.9 | -9.0 ; 5.1 | -4.6 -11.9 ; 2.7 | -4.5 -11.7 ; 2.6 | 1.5 -3.2 ; 6.3 | 1.3 -4.2 ; 6.8 | 3.0 -2.5 ; 8.6 | **8.2 2.9 ; 13.3** |

***All regression models are further corrected for: age at death, gender, educational level, partner, being religious, cancer type, metastasis at primary diagnosis and comorbidities*

**Supplement 3**: Number of total healthcare visits for each category of education and socio-economic status during the last year of life in patients with advanced cancer (n=639).


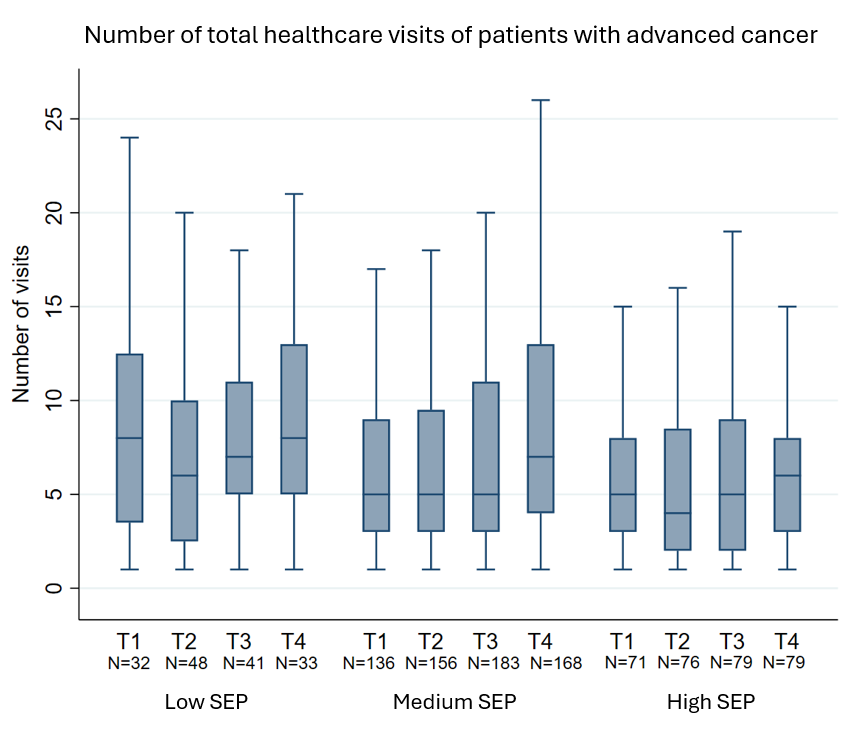


**Supplement 4:** Healthcare professionals visited in the last year of life of patients with advanced cancer (n=639) stratified by socioeconomic position and based on the questionnaire closest to death.

* Supportive care included specialist palliative care team, spiritual care, Social worker and psychologist. ** percentages do not add up to 100 as patients may have seen multiple different healthcare professionals.
